# A design metric for safety assessment of industrial robot design suitable for power- and force-limited collaborative operation

**DOI:** 10.1007/s41315-018-0055-9

**Published:** 2018-04-10

**Authors:** Bhanoday Vemula, Björn Matthias, Aftab Ahmad

**Affiliations:** 1 School of Innovation, Design and Technology, Mälardalen University, Västerås, Sweden; 2 ABB Corporate Research Center Germany, Ladenburg, Germany; 3 Department of Automation Technologies, ABB Corporate Research Center, Västerås, Sweden

**Keywords:** Collaborative robots, Safety metrics, Impact modelling, Dynamic modelling

## Abstract

This research presents a novel design metric based on maximum power flux density for the assessment of the severity of a transient physical contact between a robot manipulator and a human body region. Such incidental transient contact can occur in the course of a collaborative application of the power- and force-limiting type. The proposed metric is intended for the design and development of the robot manipulator as well as for the design of manufacturing applications. Such safety metric can also aid in controlling the robot’s speeds during manufacturing operations by carrying out rapid risk assessments of impending collisions that could arise due to the proximity to the human co-worker. Furthermore, this study contributes by expressing the physical impact between the robot and the human body region as a linear spring-damper model. The influence of the restitution coefficient and the elasticity of the human tissues on the contact duration and contact area during the collision is analysed. With the demonstrated analysis model, the dependence of the power flux density with respect to the robot’s effective mass, speed, and geometrical and damping coefficients during the human-industrial robot manipulator collision process is investigated.

## Introduction

Collaborative robot applications have gained popularity in recent times due to the ever greater need for flexibility, cost efficiency, and productivity in the manufacturing industry. In such applications, industrial robots share working space and work collaboratively together with the human co-worker. This type of manufacturing operations can harbor the risk of physical contact between robot and human. Such collisions can occur due to intended or unintended physical contact situations between them.

Whenever there is a possibility of physical contact between a robot and a human operator, the conditions of this contact must be analysed for the risk of injury in the course of the application risk assessment (Matthias et al. 2010). The mitigation of such risks is achieved by controlling the nature of these physical contacts (ISO/TS 15066 2011). This can be approached through inherently safe design measures of not only the robot mechanical systems but also of the collaborative manufacturing operations by fulfilling the safety requirements specified in international standards for robotic safety (ANSI/RIA 2012; ISO/TS 15066 2016). Such designs must limit the severity of the physical contact to a level at which the human co-worker cannot be injured and preferably experiences no pain. In this context, establishing design metrics that can quantitatively indicate the unacceptable physical impacts becomes very important and will therefore be the focus of this research.

In recent times, several researchers have carried out biomechanical investigations in order to correlate various impact quantities such as peak forces, stresses, impact displacement, force and stress impulses, kinetic energy, and energy transmitted into the human tissues, with respect to the onset of injury and pain sensations. The German Aerospace Centre (DLR) has carried out comprehensive studies in this area to analyse the impact quantities via experimental collision testing using standard automotive crash testing equipment (Haddadin et al. 2007, 2009a, b, c, 2012). In Yamada et al. (1997), experimental collision tests with live test subjects were carried out to analyse the performance limitations that must be imposed on the robot systems in order to make sure that the eventual physical contact does not lead to the onset of pain sensation. In Behrens and Elkmann (2014), mild contusions including the appearance of a bruise are established as indicators of the onset of a minor injury. Subsequently, threshold values of various impact quantities leading to contusion and onset of sensory pain were measured using experimental collision tests with live test subjects. In Unfallversicherung, D.G. (2009), specific threshold values of contact forces and pressures were introduced as relevant metrics for limiting the effects of physical contact. In Povse et al. (2010), the impact-energy density, the amount of energy transferred into the human body over a certain contact area during the course of the contact event, is used as a metric for evaluating the pain intensity. The work in Desmoulin and Anderson (2011) reports on comprehensive investigations carried out to determine various impact quantities that are required to induce a contusion in human soft tissue.

Contact forces and pressures are the impact quantities corresponding to the contact characteristics; without relation to the time they do not allow to estimate the impact severity in terms of the injury biomechanics. Therefore, the safety design metric based on maximum tensile stress as proposed in Wassink and Stramigioli (2007) cannot accurately indicate the injury biomechanics. The design metric based on energy density as reported in Povse et al. (2010) and Desmoulin and Anderson (2011) seems to be more appropriate, since transferring a certain amount of energy over a large and a small contact area turns out to have quite different impact consequences. Nevertheless, transferring a certain amount of energy over a given area in a very short time is rather more severe than transferring the same amount of energy through the same contact area over a longer period of time. Thus the relevant quantity that can best indicate impact severity seems to be power flux density. Therefore, a novel safety design metric based on power flux density will be proposed in this research study. This research also contributes by presenting a computationally efficient human–robot collision model, which can be used to quantify the proposed design metric for worst case physical collision scenarios between the robot and human body regions such as abdomen and upper limbs.

The paper is structured as follows. In Sect. 2, the safety design metric based on power flux density is introduced. In Sect. 3, a methodology for rapidly quantifying the proposed safety design metric is briefly described and subsequently verified with respect to the experimental collision data already reported in the literature, which were carried out on live human subjects at multiple human body regions. In Sect. 4, the correlation between the proposed safety design metric with respect to various human–robot collaborative workstation design variables is analysed. Finally, the conclusions are provided in Sect. 5.

## Safety design metric

The energy transferred into the human body during a physical collision with the robot systems can be defined as in (1).

1 $$ \Delta {\text{E = }}\frac{1}{2}M(V_{i}^{2} - V_{F}^{2} ), $$

where V_i_ is the relative impact velocity between the two colliding bodies just before the impact takes place, whereas V_F_ represents the relative impact velocity between the two colliding bodies just before separating from each other. The lower the value of V_F_, the greater will be the area under the force hysteresis curve and therefore the greater will be the energy absorbed by the human body. On the other hand, M represents equivalent mass, which is expressed for the unconstrained transient contact scenario, given in (2), in which the human body can retract from impact by a moving robot body. Conversely, the constrained transient contact scenario, where the human body part is constrained and cannot retract from impact by a moving robot body, is given in (3).

2 $$ M = \left( {\frac{1}{{M_{H} }} + \frac{1}{{M_{R} }}} \right)^{ - 1} $$

3 $$ {\text{M}} = {\text{M}}_{\text{R}} , $$

where M_H_ and M_R_ are the effective masses of the human and robot bodies, respectively. Since the ratio of V_F_ and V_i_, which is commonly termed as restitution coefficient (C_R_), gives the measure for the kinetic energy loss during the impact, Eq. (1), can be parametrized in terms of C_R_ as (4) (Hunt and Crossley 1975).

4 $$ \Delta {\text{E = }}\frac{1}{2}MV_{i}^{2} ( 1- C_{R}^{2} ) $$

In general, the safety assessments during the design phases are carried out for the worst-case scenario, which can be characterized by assuming that all the kinetic energy from the robot system is transferred into the human body. This assumption can be expressed in (4), by considering the value of C_R_ to be closer to zero. From the work energy principle, energy transferred can be expressed in terms of work done as given in (5) (Povse et al. 2010).

5 $$ \Delta {\text{E = }}\mathop \smallint \limits_{0}^{{\delta_{ \hbox{max} } }} F_{C}\,   {\text{d}}\delta $$

Finally, the power flux density (W), which represents the energy transferred over a certain contact area (A_C_) over a period of time, can be expressed as in (6).

6 $$ {\text{W = }}\frac{{ F_{c} \dot{\delta }}}{{A_{C} }} , $$

where F_C_ represents the contact force due to the impact between the robot and human bodies and $\dot{\delta }$ represents the rate of deformation during the contact process. However, in practice, the physical impact between the robot and the human body is assumed to be completely inelastic for the worst-case scenario, where the kinetic energy is directly used in energy density calculation. The influence of the elastic properties of the human body regions will not be considered for energy calculation. But these properties need to be considered to calculate the contact area and the rate of contact deformation of the human soft tissues for power flux density estimation. Therefore, in the next section a contact force model is presented which can be used to estimate the impact quantities required to estimate the power flux density values.

## Impact model between robot and human body regions

The normal contact process between robot and human bodies is expressed as a single-degree-of-freedom dynamic system. When the contact process begins, the local deformation between the robot and human bodies is zero and the contact velocity is given by (V_Robot_ − V_Human_); these initial conditions can be used for expressing the contact force F (δ) using the equation of motion in terms of the local deformation (δ) (7).

7 $$ \ddot{\delta } = \frac{F(\delta )}{M} $$

**Fig. 1:**
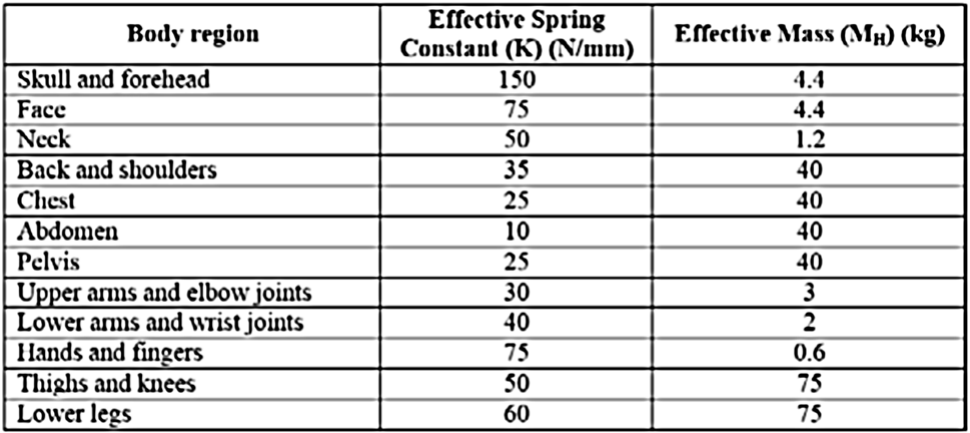
Averaged effective masses (Haley 1988) and spring constants (Unfallversicherung, D.G. 2009) of different human body regions

Depending on the type of transient contact case, M is defined as a function of M_R_ and M_H_ based on (2) and (3). For a given multi-link robot manipulator, M_R_ at the point of impact on the robot body in the operational space can be accurately calculated for a specific set of joint space parameters (q) from the kinetic energy matrix M_C_ in the operational space (x) as given in (8) (Khatib and Burdick 1987). Based on the decomposition of the kinetic energy matrix inverse, the robot’s reflective mass (M_R_), which is a scalar value, can be obtained. M_R_ is the robot’s mass perceived at the end effector given a force in u direction as given in (8) (Khatib and Burdick 1987).

8 $$ \begin{aligned} {\text{M}}_{{\text{C}}} &= ({\text{J(q)M}}^{- 1} ({\text{q)J}}^{{\text{T}}} ({\text{q}}))^{- 1}  \\ {\text{M}}_{{\text{R}}} ({\text{x}}) &= [{\text{u}}^{{\text{T}}} {\text{M}}_{\text{C}}^{ - 1} ( {\text{q}}){\text{u}}]^{ - 1} , \end{aligned} $$

where J(q) is the Jacobian matrix and M(q) is the symmetric positive definite mass matrix. u is the unit vector. On the other hand, data related to the M_H_ values corresponding to different body regions are adopted from Haley (1988) and are shown in Fig. 1 for reference. These are averaged mass values collected from different human subjects of various age groups, genders, and anthropometric diversity.

The physical impact between the robot and human bodies can be expressed as a linear spring-damper system, which can be used as a simplified modeling approach to represent the physical nature of the energy transferred between them during the contact process. In such models, contact force can be represented as a contact force law with a linear viscous-elastic term as shown in (9) (Hunt and Crossley 1975; Hertz 1881).

9 $$ {\text{F = K}}\delta {\text{ + C}}\dot{\delta }\delta , $$

where K represents the contact stiffness, C is the damping coefficient, and $\dot{\delta }$ is the rate of deformation during the impact process. Several researchers have proposed various expressions for C as a function of C_R_ (Hunt and Crossley 1975; Flores et al. 2006), resulting in many variants of contact force models, which can account for dissipative forces. The proposed study aims at evaluating the highly inelastic collisions for the worst-case considerations. Therefore, the formulation as given in (10) for expressing the damping coefficient is adopted from Flores et al. (2006), since it has been demonstrated to be reliable for the impact cases with smaller C_R_ values.

10 $$ {\text{C = }}\frac{{ 8 {\text{K(1}} - C_{R} )}}{{5C_{R} V_{i} }} $$

From (9) and (10), the contact force (F_C_) can be expressed as (11).

11 $$ F_{C} {\text{ = K}}\delta \left[ { 1 { + }\frac{{ 8 ( 1- C_{R} )}}{{5C_{R} }}\frac{{\dot{\delta }}}{{V_{i} }}} \right], $$

where K represents the effective spring constant corresponding to a specific human body region. The values of K for different body regions are experimentally derived for different body regions in Unfallversicherung, D.G. (2009), which can be referred to in Fig. 1. These compression constants corresponding to different body regions are averaged values measured from several human subjects of different genders and anthropometric diversity. These data are currently used in the force- and pressure-measuring devices intended to carry out human–robot collision-based risk analysis (Dagalakis et al. 2016; Huelke and Ottersbach 2012). By integrating (7) at several points of time during the collision process, the local contact deformation (δ) and the rate of deformation $\dot{\delta }$ can be calculated, which can subsequently be used to estimate the contact area (A_C_) by using the expression (12) (Johnson 1987). It could be noted that the area of contact is assumed to be circular in this case.

12 $$ A_{C} \,{ = 2}\, \pi \delta R_{C} $$

Finally, by substituting F_C_, A_C_, and $\dot{\delta }$ in (6), the impact severity measure based on power flux density (W) can be determined. It is of significance that the contact model presented in this section should be able to estimate the relevant impact quantities with reasonable accuracy in order to arrive at a reliable value of W. Therefore, in this study, the impact quantities estimated from the presented rapid contact model (RCM) are subjected to a verification process by comparing them from the experimental collision tests on live human subjects reported by Behrens and Elkmann (2014). The rest of this section presents a brief overview of the experimental setup and collision test data adopted from Behrens and Elkmann (2014). These data are subsequently used in this research to verify the proposed RCM.

### Collision tests on live human subjects

The experimental setup used by Behrens and Elkmann (2014) to conduct the collision tests is based on a coupled pendulum hanging from a stiff frame. A stiff rack was installed in front of the frame to fasten different body parts of the human subjects before inducing the blunt impacts with an impactor.

The inertia and the damping coefficient of the pendulum were determined by fitting a model of the pendulum on the measurement acquired in a specific oscillation experiment. This experimental setup yielded two masses, 4.16 and 8.65 kg, respectively, by varying the effective mass of the pendulum by means of a mounting device (Behrens and Elkmann 2014). These masses are typically representative of the reflective masses observed in small collaborative robots like LWR of the DLR (Haddadin et al. 2009). Subsequently a piezo-electric load cell was used to measure the impact forces, whereas the pendulum angle deflection is measured by a precise potentiometer during the collision tests on the live test subjects.

### Experimental collision test data

The research reported in Behrens and Elkmann (2014) carried out collision tests on three different locations on the left arm (lower arm, upper arm, and shoulder). These parts were chosen due to their high likelihood of being involved in a collision during a collaborative application. Furthermore, since the biomechanical properties of the body regions vary considerably among different human subjects depending on their gender and other anthropometric variations, the collision tests were carried out on four human subjects. Subsequently, collision data in terms of impact forces at different time steps were measured for 17 different collision experiments. These collision experiments were designed by varying the human subject, location of impact on the human arm, and effective mass and velocity of the impactor, since the contact model formulation presented in this paper makes use of the averaged stiffness properties of the human body regions. Therefore, it can be justified that outputs from the contact model be verified from experimental collision test data from any one specific human subject. Therefore in the research the first five collision experiments reported in Behrens and Elkmann (2014) will be exploited to verify the RCM presented in this study. The data from these five experimental collision tests were applied to subject 1 at location 1 and location 2, which refers to the human subject’s lower arm and upper arm regions, respectively. These five experiments also include different combinations of the impact masses and velocity as can be seen in Table 1. The peak force (F_max_) values are taken from the tabular data provided in Behrens and Elkmann (2014), whereas the time duration to reach the peak forces (T_P_) was visually observed from the published graphs and therefore approximated in this study for the purpose of verification.

**Table 1:** Experimental collision data from Behrens and Elkmann (2014)

#	Subject	Location	Mass (kg)	Impact velocity (m/s)	F_max_ (N)	T_P_ (ms)
1	1	1	8.65	0.64	351	~ 28
2	1	1	8.65	0.74	447	~ 26
3	1	2	8.65	0.83	393	~ 31
4	1	2	8.65	0.94	523	~ 28
5	1	2	4.16	0.83	212	~ 22

### Verification process

The simulations based on RCM are set up by assigning the robot’s mass (M_R_) and impact velocity (V_i_) according to the parameters defined in Table 1.

The biomechanical stiffness of locations 1 and 2 is defined as 40 and 30 N/mm, respectively, which corresponds to the averaged linear stiffness values of human lower and upper arm as in Fig. 1.

**Fig. 2:**
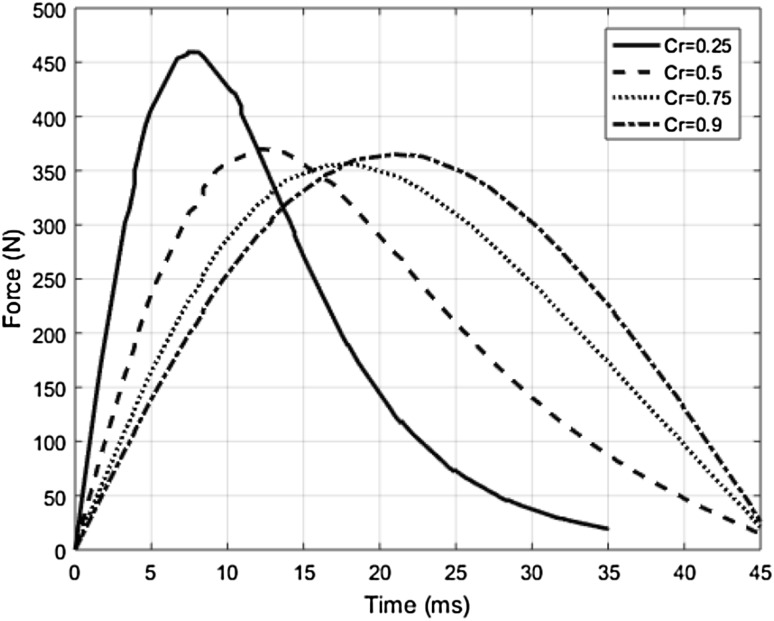
Time response of the contact forces corresponding to Experiment 1 for different C_R_ values

Finally, in order to complete the contact model formulation, the value of the restitution coefficient (C_R_) in (11) needs to be assigned. To tune the value of C_R_, experiment 1 from Table 1 is simulated using the RCM model by assigning four different values of C_R_ as represented in Fig. 2. It can be observed that the peak contact forces and the time taken to reach these peak forces correlate to a better extent with respect to the experimental data when the Cr value is 0.9. Further, from the visual observation of the force–time response graphs of the collision experiments reported by Behrens and Elkmann (2014), it can be noticed that the time taken to reach full compression and relaxation during the collisions with lower and upper arm is the same. Therefore, it can be assumed that the experimental collision cases were more elastic in nature. Hence, C_R_ values of 0.9 can be used in order to verify the RCM model with respect to the experimental collision data reproduced in Table 1.

**Fig. 3:**
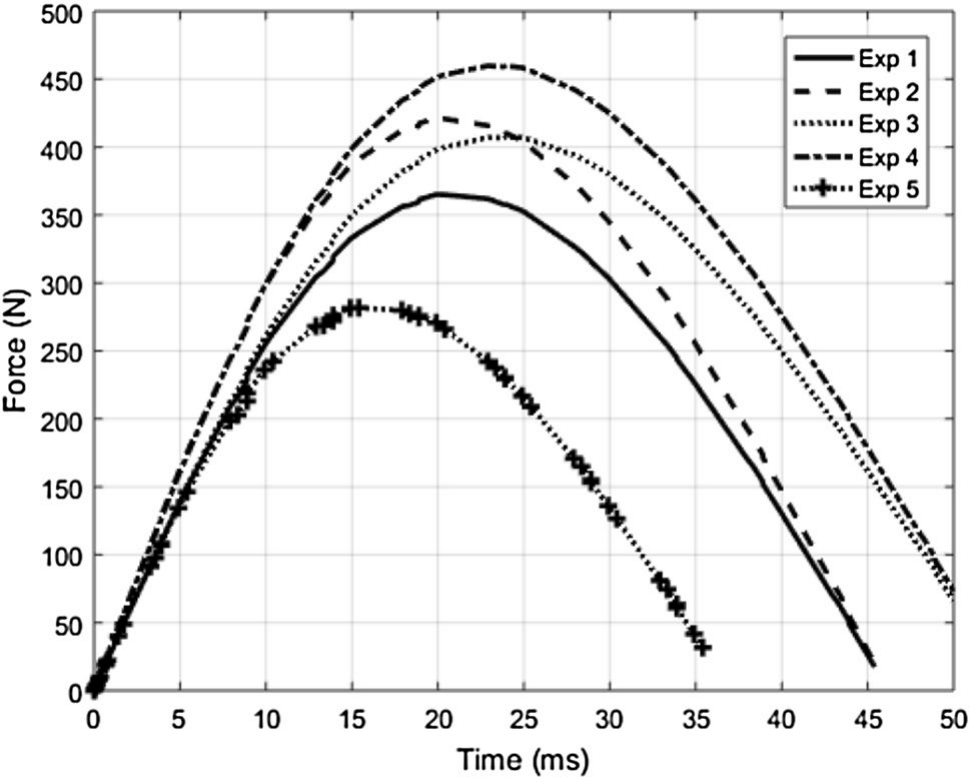
Time responses of contact forces from the RCM model corresponding to the input parameters from experimental collision data given in Table 1. Experimental collision data from Behrens and Elkmann (2014)

Constrained transient collisions between the robot and human body regions according to Table 1 are simulated by using the RCM model by assuming the C_R_ to be 0.9 (based on the analysis from Fig. 2). The resulting contact forces at different points of time during the impact can be seen in Fig. 3. Subsequently the peak forces and time taken to reach the peak forces measured from the RCM models are compared with respect to those from the experimental collision data (EXP) in Table 2. It can be noted from the comparison that F_max_ and T_P_ values from the RCM deviate at an average of about 9 and 18%, respectively, from those from the experimental collision tests. It can be noted that this deviation could be due to many factors such as the variation of the biomechanical stiffness properties of the human subject from the averaged value, the choice of the C_R_ tuned in the RCM model, or the simplification of the contact force formulation in the RCM model.

**Table 2:** Peak contact forces (F_max_) and contact duration to reach F_max_ (T_P_) from RCM and experimental collision data

#	F_max__(N) (RCM)	F_max__(N) (exp)	T_P_ (ms) RCM	T_P_ (ms) (exp)
1	360	351	22	~ 28
2	420	447	22	~ 26
3	410	393	25	~ 31
4	460	523	24	~ 28
5	280	212	17	~ 22

However, considering the limitations in the currently available analytical modeling approaches in the literature to realistically represent the highly nonlinear, isotropic, and non-homogeneous nature of the physical collisions, this deviation of the model can be considered reasonable for the purpose of rapidly evaluating the severity of human–robot collisions during a collaborative manufacturing operation. Therefore, the relative agreement of F_max_ as well as T_P_ calculated from the RCM model with respect to the experimental collision data can be used as a measure for the reliability of the proposed rapid contact model for the purpose of estimating the power flux density values.

## Influence of human–robot collaborative workstation design parameters

**Fig. 4:**
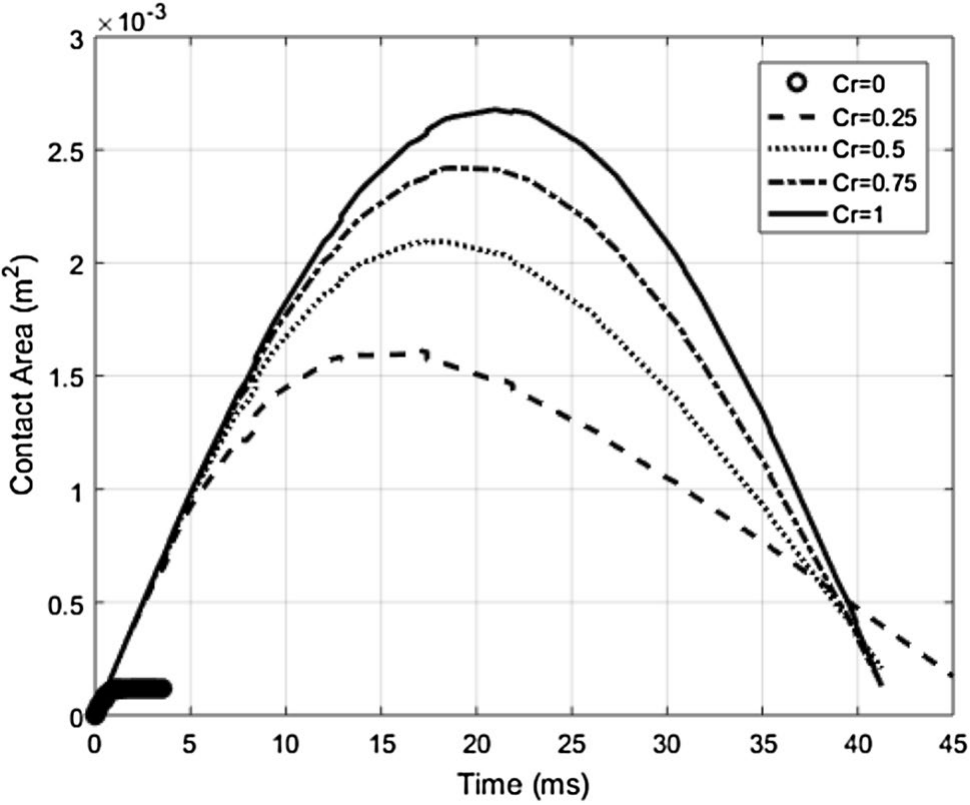
Resulting contact area at different points of time during the collision process as a function of restitution coefficient (C_R_)

Various impact quantities such as contact area, contact forces, rate of local deformation, and the amount of energy deposited, show a high degree of sensitivity with respect to the restitution coefficient (C_R_). For instance, the contact area and contact duration during the collision process between the robot and human lower arm region for the experimental collision case 1 (Table 1) can be seen in Fig. 4. It can be observed that, as the value of C_R_ approaches zero, the amount of energy transferred will increase considerably over a smaller and smaller contact area in a shorter duration of time. Thus a truly conservative design metric in terms of power flux density will be reached at a purely inelastic impact scenario (C_R_ → 0), in which case all the kinetic energy of the robot system will be deposited into the human body. Therefore, a C_R_ value in the range of 0.2–0.5 can be recommended for design evaluation purposes, since setting the C_R_ value closer to zero might lead to complexity in meeting the design constraints, which can limit the robot performance to an extent where it might be difficult to execute the human–robot collaborative manufacturing operations.

Further, from the robot side, the main impact parameters are its reflective mass, impact velocity, and the robot surface curvature.

Therefore, as shown by the DLR, in a collision, a complex robotic system can be reduced to a rigid object that can be characterized by the parameters velocity (V_i_), robot mass (M_R_), and robot surface shape (R_C_) (Haddadin et al. 2012).

Therefore, the rest of this section presents an analysis that is carried out by arbitrarily varying these parameters along with the biomechanical stiffness properties of the human subjects in order to demonstrate their influence and correlation with respect to the safety design metric proposed in this study.

### Influence of robot mass (M_R_) and impact velocity (Vi)

For this sensitivity analysis, the value of C_R_ is set at 0.2. Subsequently, maximum values of the power flux density are measured during constrained transient collisions between the robot and the human’s upper arm region which has an averaged stiffness value of 30 N/mm.

**Fig. 5:**
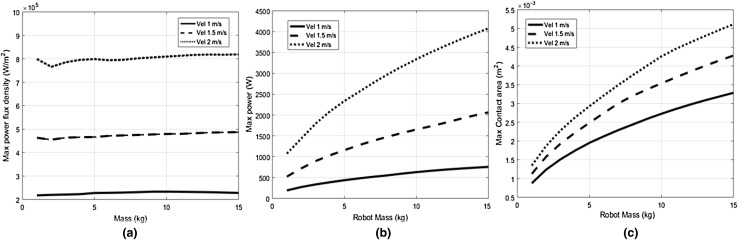
**a** Resulting maximum power flux density as a function of robot mass at different impact velocities. **b** Resulting maximum power as a function of robot mass at different impact velocities. **c** Resulting maximum contact area as a function of robot mass at different impact velocities

In general, the robot’s effective mass and impact velocities have an incremental effect on the kinetic energy transferred and are therefore expected to have a considerable influence on the maximum power flux density (W) values. However, from Fig. 5a it can be observed that only the impact velocity of the robot has shown to influence W. On the other hand, the variation of robot mass has not resulted in the variation of W, even though increase in robot mass yielded higher maximum power values (by means of energy deposition and lowered contact duration), which is evident from Fig. 5b The increase in robot mass resulted, however, in higher strain energy, thereby increasing the impact penetration, which consequently increased the maximum contact area as shown in Fig. 5c. Therefore, eventually the increase in maximum power is compensated by the increase in the maximum contact area, thereby resulting in the saturation of W with the variation of robot mass.

From this analysis it can be concluded that both maximum power values and contact area need to be used as safety measures in order to correlate the injury severity with the variation of robot mass.

### Influence of the robot’s radius of curvature (R_C_)

**Fig. 6:**
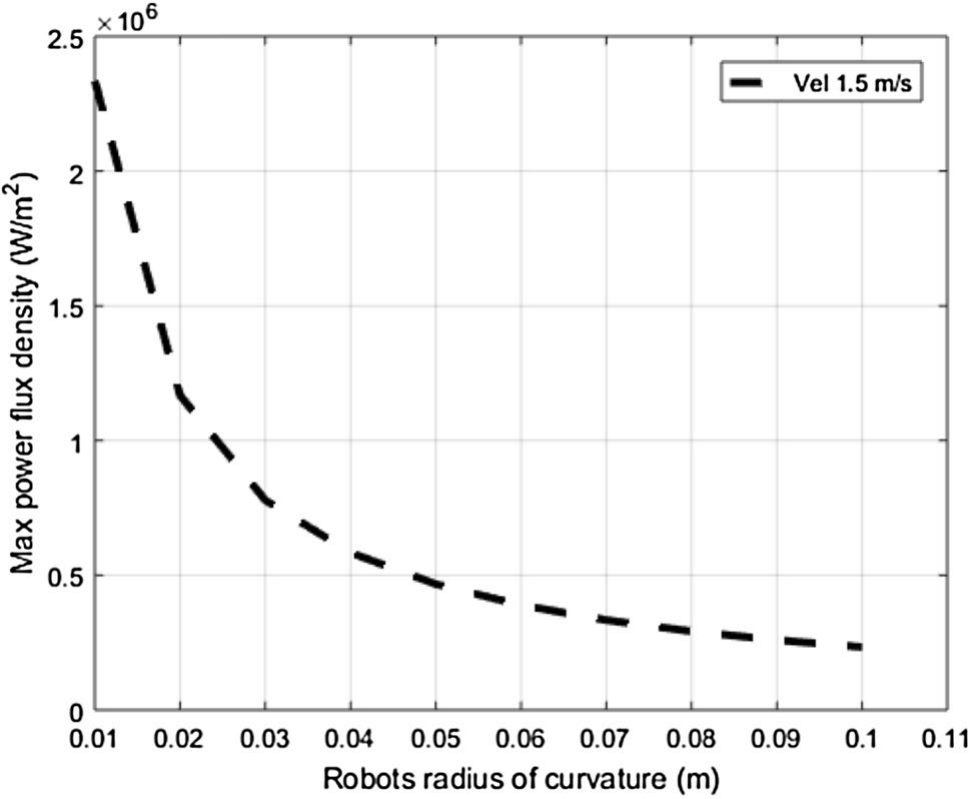
Resulting maximum power flux density values as a function of the robot’s radius of curvature (R_C_)

For this sensitivity analysis, the values of C_R_, M_R_, and V_i_ are set at 0.2, 10 kg, and 1.5 m/s, respectively. Subsequently, maximum values of the power flux density are measured during constrained transient collisions between the robot and the human’s upper arm region, which has an averaged stiffness value of 30 N/mm. The robot’s radius of curvature also substantially influences the contact surface area as given in Eq. (12). Therefore, the increase in the robot’s radius greatly affects the maximum power flux density values as can be seen in Fig. 6; the larger radius of curvature can surely be accommodated by considering thicker padding material in combination with the right degree of elasticity.

However, this may sometimes be counterproductive from a safety point of view of the human’s risk perception due to the increased size of the robot arm.

### Influence of the human’s stiffness properties (K)

**Fig. 7:**
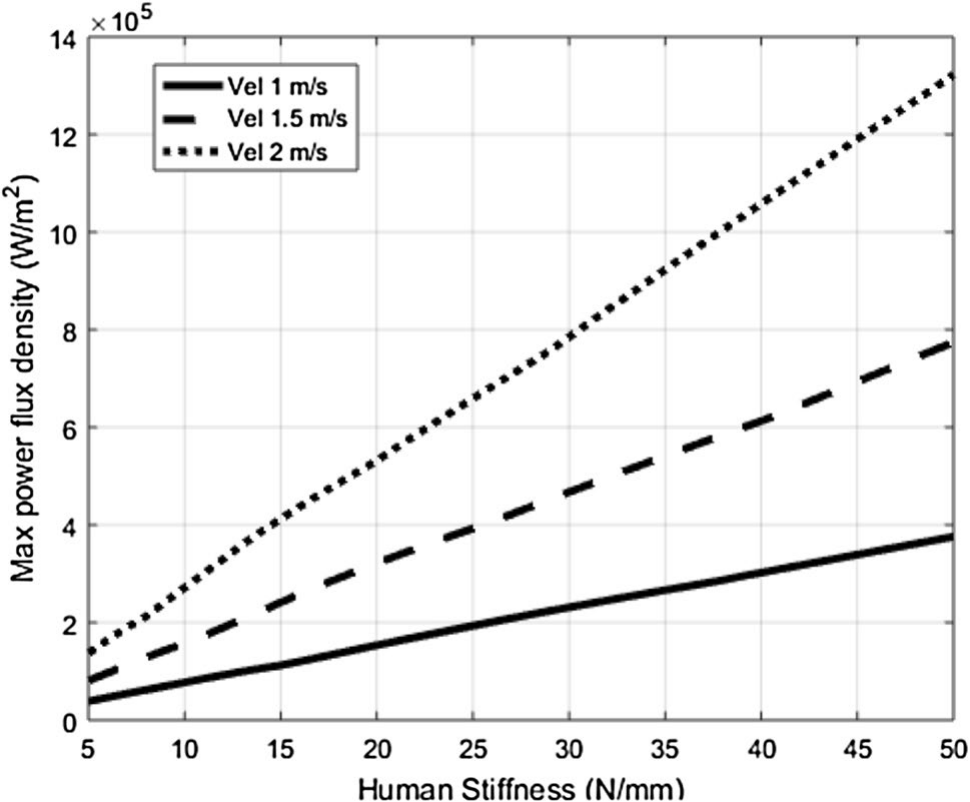
Resulting maximum power flux density values as a function of the human stiffness properties (K)

For this sensitivity analysis, the values of C_R_ and M_R_ are set at 0.2 and 10 kg, respectively. Subsequently, maximum values of the power flux density are measured during constrained transient collisions between the robot and the human body with different stiffness properties (K). The increased K values will lead to a considerable increase in the energy deposition and a decrease in the contact area during the collision process. Therefore, as can be seen in the Fig. 7, an increase in K values showed higher maximum power flux density values.

From this analysis it can be concluded that the safety measure based on power flux density has shown a consistent and expected correlation with the variation of the most important collision parameters such as the impact velocities, the robot’s surface, and stiffness properties.

## Conclusions and discussion

The contribution from this study is twofold. First, a safety design metric based on power flux density is introduced, which can take into account impact quantities such as magnitude of energy transfer, contact area, and contact duration in order to come up with a more inclusive indicator for the occurrence of contusion or onset of pain sensation during an event of impact between the robot and the human body. Second, this study contributes by expressing the physical impact between the robot and the human body as a linear spring-damper model and analysing the effect of restitution coefficient and elasticity of the human body regions on the contact duration and contact area during the collision process. With such an analysis model, a correlation of the power flux density with the robot’s speed, geometrical properties of the robot’s surface, and biomechanical stiffness properties of human body parts has been demonstrated. However, safety measures based on power and contact area need to be individually considered to establish the correlation of the robot’s reflective mass with respect to injury severity.

As of now, there is no clear view in the research community as to what impact quantity can be most relevantly used as an indicator for the occurrence of contusion or pain sensation in the human body. However, this research gap is expected to be addressed by the ongoing biomechanical research in the coming years (Behrens and Elkmann 2014). In hindsight, a general methodology for quantifying all the relevant impact quantities will be very useful for the robotics community, especially during the design and development stages of robot systems and their applications. Therefore, the safety design metric proposed in this research can be used as a design criterion that needs to be minimized for the purpose of designing safer robot mechanical systems and collaborative manufacturing operations, without necessarily trying to go all the way in predicting the threshold values of the power flux density values for the onset of contusions or pain sensations experienced by humans.

Nevertheless recent work has been conducted in application of energy criteria in the control of power and force limited manipulators (Geravand et al. 2016; Meguenani et al. 2015; Rossi et al. 2015). In a future work, one might consider replacing the energy criterion by power flux density limits on prospective robot–human contacts. While this would still result in adaptation of the joint speed, the determination of contact area and contact duration as part of the power flux density criterion remains. Assuming we do not have real time information on the immediately exposed body region, these quantities need to be determined beforehand for contact cases identified in the risk assessment of the application. In particular, data describing surface material and geometry of the manipulator as well as visco-elastic properties of the relevant body regions enter into this computation.

Furthermore, it can be noted that for the collision cases studied, the effective stiffness corresponding to that of the human’s lower and upper arm regions is much lesser than that of the robot. Therefore, the elasticity of the robot did not show any considerable influence on the contact area, since in essence the effective stiffness of both the robot and the human body region acts as if in a series and the resultant contact stiffness values eventually round off towards the compression constant corresponding to that of the human lower and upper arm. On the other hand, when collision scenarios arise with somewhat stiffer body regions, then the softness of the robot will have a considerable influence in distributing the dissipated energy over a larger contact area, consequently decreasing the value of power flux density. Such behavior is highly desired from a safety point of view and therefore needs to be considered during contact modeling. However, since the contact model presented is mainly intended to evaluate worst-case scenarios during human–robot collaborative manufacturing application design, the inherent safety potential of the robot system due to its softness is ignored. Nevertheless, ignoring the influence of the robot’s elasticity is one of the major limitations of the proposed contact model. This issue is planned to be considered in greater depth in our future work in order to fully capture the collision behavior between the robot and human body regions.
